# Patient years lost due to cytomegalovirus serostatus mismatching in the scientific registry of transplant recipients

**DOI:** 10.3389/fimmu.2023.1292648

**Published:** 2024-01-09

**Authors:** Maheen Z. Abidi, Jesse D. Schold, Bruce Kaplan, Adriana Weinberg, Kristine M. Erlandson, John S. Malamon

**Affiliations:** ^1^ Department of Medicine, Division of Infectious Diseases, University of Colorado, Aurora, CO, United States; ^2^ Department of Surgery, Division of Transplant Surgery, University of Colorado, Aurora, CO, United States; ^3^ Colorado Center for Transplantation Care (CCTCARE), Research and Education, Division of Transplant Surgery, Department of Surgery, Anschutz Medical Campus, University of Colorado, Aurora, CO, United States; ^4^ Department of Pediatrics and Pathology, University of Colorado, Aurora, CO, United States

**Keywords:** CMV, serostatus, survival, graft, kidney, transplant

## Abstract

**Background:**

The cytomegalovirus (CMV) mismatch rate in deceased donor kidney transplant (DDKT) recipients in the US remains above 40%. Since CMV mismatching is common in DDKT recipients, the cumulative effects may be significant in the context of overall patient and graft survival. Our primary objective was to describe the short- and long-term risks associated with high-risk CMV donor positive/recipient negative (D+/R-) mismatching among DDKT recipients with the explicit goal of deriving a mathematical mismatching penalty.

**Methods:**

We conducted a retrospective, secondary analysis of the Scientific Registry of Transplant Recipients (SRTR) database using donor-matched DDKT recipient pairs (N=105,608) transplanted between 2011-2022. All-cause mortality and graft failure hazard ratios were calculated from one year to ten years post-DDKT. All-cause graft failure included death events. Survival curves were calculated using the Kaplan-Meier estimation at 10 years post-DDKT and extrapolated to 20 years to provide the average graft days lost (aGDL) and average patient days lost (aPDL) due to CMV D+/R- serostatus mismatching. We also performed an age-based stratification analysis to compare the relative risk of CMV D+ mismatching by age.

**Results:**

Among 31,518 CMV D+/R- recipients, at 1 year post-DDKT, the relative risk of death increased by 29% (p<0.001), and graft failure increased by 17% (p<0.001) as compared to matched CMV D+/R+ group (N=31,518). Age stratification demonstrated a significant increase in the risk associated with CMV mismatching in patients 40 years of age and greater. The aGDL per patient due to mismatching was 125 days and the aPDL per patient was 100 days.

**Conclusion:**

The risks of CMV D+/R- mismatching are seen both at 1 year post-DDKT period and accumulated throughout the lifespan of the patient, with the average CMV D+/R- recipient losing more than three months of post-DDKT survival time. CMV D+/R- mismatching poses a more significant risk and a greater health burden than previously reported, thus obviating the need for better preventive strategies including CMV serodirected organ allocation to prolong lifespans and graft survival in high-risk patients.

## 1 Introduction

Cytomegalovirus (CMV) has a seroprevalence rate of 50% in the United States; seroprevalence increases with age (1, 2) and remains an important cause of morbidity and mortality in solid organ transplant (SOT) recipients (3–5). Following SOT, latent CMV virus can reactivate in CMV seropositive recipients (CMV R+). SOT recipients with a CMV seronegative status (CMV R-) who receive organs from CMV seropositive donors (CMV D+) are at the highest risk of developing primary CMV infection and disease (6), typically during the first three months following SOT (7). Compared to CMV R+ who have at least some level of CMV immunity at the time of SOT, CMV D+/R- SOT recipients are immunologically naïve and may have an impaired immune response in the setting of post-transplant immune suppression, with impaired cell-mediated immune responses (8). The development of CMV-specific, cell-mediated immunity has been associated with protection against clinically significant CMV infection (8, 9). In addition to infection in the first three months following SOT, delayed onset CMV disease may occur in patients following the cessation of CMV prophylaxis (10). Furthermore, CMV has several indirect effects given its ability to serve as an immunomodulator. It has been implicated in acute rejection, chronic allograft injury (5, 11, 12), coronary vasculopathy in heart transplant recipients (13–15), and bronchiolitis obliterans in lung transplant recipients (16). While the exact mechanisms of these conditions remain unclear, CMV is thought to contribute to a proinflammatory condition that accompanies aging, also known as “inflammaging” (17).

Other studies have noted the risks associated with high-risk CMV D+/R- mismatching in deceased donor kidney transplant (DDKT) recipients (18–20), with a consensus that CMV mismatch poses a significant risk to DDKT recipients. However, no prior studies have investigated the penalty of CMV D+/R- mismatch across the lifespan of a DDKT recipient or in the DDKT population at large. The main objectives of this study were: 1) to utilize the Scientific Registry of Transplant Recipients (SRTR) to provide an accurate and comprehensive analysis of the short-term, long-term, and cumulative effects of CMV mismatching in a donor-paired DDKT population, and 2) to determine differences in these effects by age, while adjusting for differences in donor and recipient characteristics. Since the risks associated with CMV mismatching are accumulated throughout the patient’s lifespan and aggregate across the entire mismatched population, we developed and calculated two metrics to underscore the magnitude of CMV D+/R- mismatching and bring this most important problem into context.

## 2 Materials and methods

### 2.1 Data sources

This study utilized data from the SRTR database. SRTR includes data on all donors, waitlisted candidates, and transplant recipients in the US, submitted by the members of the Organ Procurement and Transplantation Network (OPTN) (21). The Health Resources and Services Administration, U.S. Department of Health and Human Services provides oversight to the activities of the OPTN and SRTR contractors. The data reported here have been supplied by the Hennepin Healthcare Research Institute as the contractor for SRTR. The interpretation and reporting of these data are the responsibility of the author(s) and in no way should be seen as an official policy of or interpretation by the SRTR or the U.S. Government.

### 2.2 Study ethics

Informed consent was obtained for all study participants. This study was reviewed by an ethical committee (Colorado Multiple Institutions Review Board) and was determined to be non-human subject research. The authors do not have any relevant conflicts of interest to disclose. All authors reviewed the results and approved the final manuscript.

### 2.3 Inclusion criteria

Characteristics at admission for DDKT recipients were extracted from the SRTR database, including age, sex, race, ethnicity, pre-dialysis (yes/no), diabetes mellitus (yes/no), creatinine at the time of DDKT, the total number of recipient Human Leucocyte Antigen (12) mismatches, and the total number of days spent on the waitlist. This study population included adult DDKT recipients with an initial transplant between January 1, 2012, and September 2, 2022. Multi-organ transplant recipients and those undergoing re-transplantation were excluded. P-values were provided via the Analysis of Variance (ANOVA) test for continuous variables and the chi-squared method was used for testing the significance of indicator and categorical variables. Recipients with missing data were removed prior to analysis. The race and ethnicity of study participants were self-reported and recorded by SRTR.

### 2.4 Paired kidney analysis

**Figure 1:**
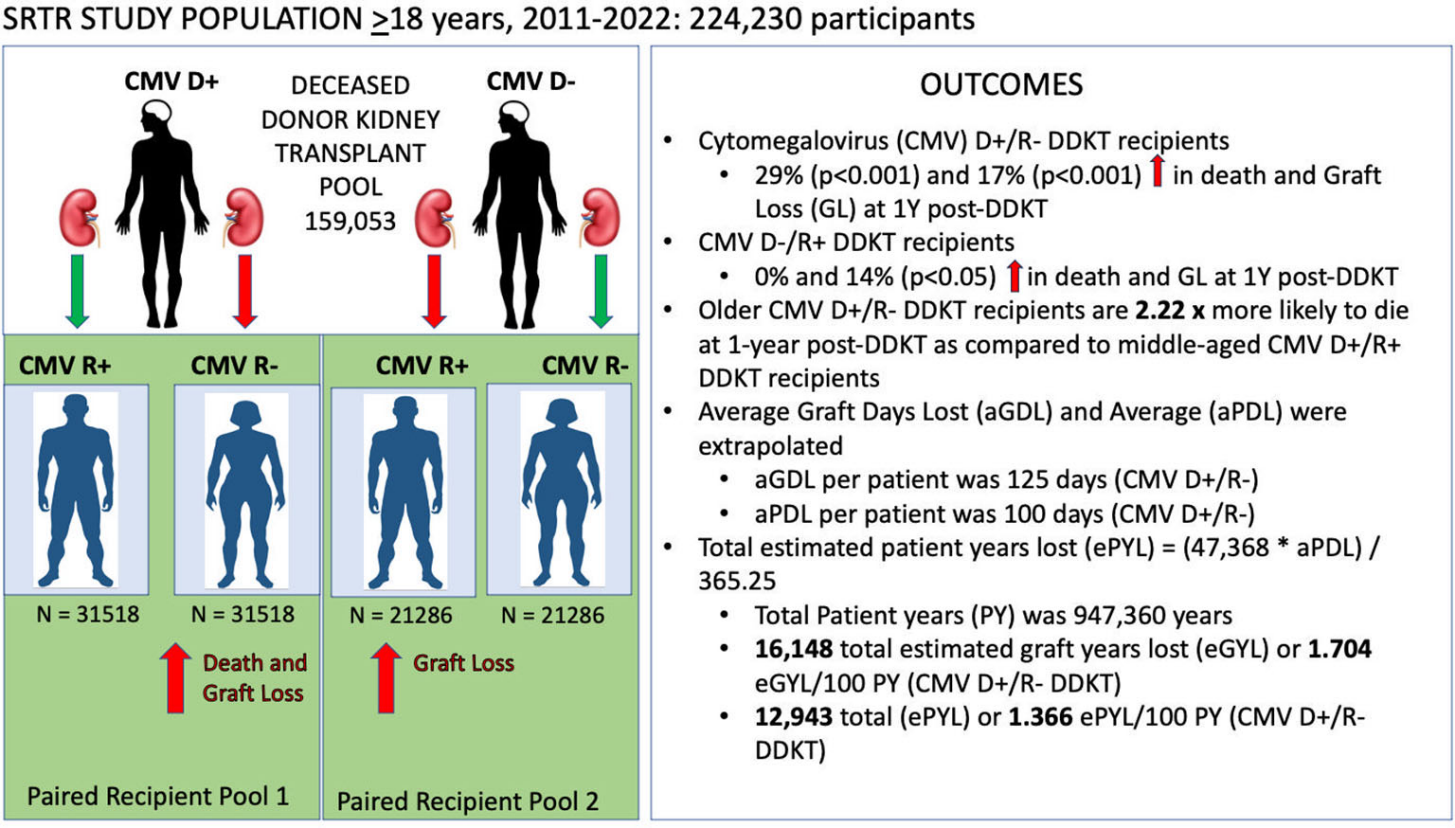
Graphical abstract of paired-deceased donor kidney CMV mismatch study design. This graphical abstract illustrates our study design (left panel) and the main outcomes (right panel). Here we illustrate our donor-paired kidney analysis, where we matched 31,518 CMV donor-positive (CMV D+) recipient pairs and 21,286 CMV donor-negative (CMV D-) recipient pairs. Risk ratios and survival curves were independently calculated for each CMV donor positive/recipient positive (CMV D+/R+) and CMV donor negative/recipient negative (CMV D-/R-) cohort from 1 year post-deceased donor kidney transplant (DDKT) to 10 years post-DDKT. CMV, cytomegalovirus; SRTR, Scientific Registry of Transplant Recipients.

To normalize biases resulting from non-random differences in the characteristics of the donor population, we employed a paired kidney analysis by only including kidney donors with two recipients. We identified a total of 105,608 donor-paired DDKT recipients. Each recipient pool was matched to only contain individuals who received one kidney from the same donor and had an opposite CMV serostatus. The CMV D+ paired- recipient group included CMV R+ (N=31,518) and CMV R- (N=31,518), and the CMV D- paired- recipient group included CMV R+ (N=21,286) and CMV R- (N=21,286). Each donor-paired group (CMV D+, CMV D-) was analyzed independently. **Figure 1** provides a graphic abstract of this study design.

### 2.5 Statistical approach

From the donor’s perspective, CMV serostatus matching was handled in two manners: 1) match to a recipient with the same serostatus, or 2) mismatch to a recipient with the opposite serostatus. To quantify the effects of CMV mismatching, we analyzed the total CMV D+ (N=63,036) and CMV D- (N=42,572) cohorts independently to measure any differences in all-cause graft failure and mortality, ranging from 1 year (1Y) to 10 years (10Y) post-DDKT. We then measured the relative short- and long-term effects of CMV mismatching in each paired cohort. The CMV serostatus at the time of DDKT was defined as CMV+ if the CMV immunoglobulin (IgG) antibody was recorded as positive and defined as CMV- if the CMV IgG antibody was recorded as negative in SRTR. Prior to analysis, we excluded 2.5% of patients with missing values for donor or recipient CMV serostatus. Less than 5% of the total donor paired DDKT recipients had one or more values missing and were removed prior to all analyses.

### 2.6 Risk and survival analysis

Censoring times for DDKT recipients started at the date of transplant and were censored at the date of graft loss, death, and the date of the last administrative follow-up. If the follow-up date was missing, but the patient was alive, September 2, 2022, was used as the date of the last administrative follow-up. We examined short- and long-term outcomes ranging from 1 year to 10 years post-DDKT. The adjusted hazard ratios (HRs) were calculated using the Cox proportional-hazard regression (CPHR) analysis and were adjusted for the recipient’s age at the time of transplantation, sex, race, ethnicity, diabetes status, the total number of HLA mismatches present at the time of transplant, whether the recipient had received dialysis prior to transplantation, and total creatinine at the time of transplantation. The CPHR model is a semi-parametric regression method that models the relationship between events, survival times, and one or more independent variables or covariates (22). To independently measure the effects of the DDKT recipient’s age in association with CMV mismatching, DDKT recipients were grouped according to three age classifications: young (18-40 years), middle-aged (41-64 years), and older (≥ 65 years). To further examine patient risk factors, we stratified by diabetes and dialysis status and reported all hazard ratios. We then calculated hazard ratios for each age group using the CPHR model. All survival probability curves were generated using the non-parametric Kaplan-Meier (KM) (23) estimation. KM curves were generated for all cohorts using the same covariates described above. Because the goal of this study was to also provide a cumulative patient and population CMV mismatch penalty, the average patient days (aPDL) lost due to mismatching was empirically measured up to 10 years post-transplant by calculating the difference in the cumulative sum of the area under the KM curves (AUC) between the CMV D+/R+ and CMV D+/R- groups. Here the cumulative sum of risk is defined as the integrated sum of the probability of an event in time. We leveraged the rmst2 package in R to calculate the AUC of all KM curves (24). This approach was applied to graft loss KM curves to determine the average graft days (aGDL) lost due to mismatch.

### 2.7 Extrapolation using parametric survival regression

Finally, we applied parametric survival regression (25) assuming a log-normal distribution to extrapolate the average graft days lost and average patient days lost up to 20 years post-transplant for the CMV donor-positive cohort. The median graft survival for a deceased donor kidney has been projected at 19.2 years in 2021 (26). We have previously used this approach to accurately estimate the survival benefit of a liver transplant over a patient’s natural lifespan (27, 28). Extrapolation was performed with the variables at the time of transplant and the 10 years follow up period. We calculated the aGDL and aPDL lost at 20 years by subtracting the AUC of the two extrapolated KM curves, CMV D+/R- and CMV D+/R+. The aGDL, and aPDL lost were then used to estimate the aggregate effects of CMV mismatching in this population using linear approximation. To do this, we multiplied the total number of paired CMV D+ mismatches (N=47,368) observed in this study period by the average CMV D+ mismatch penalty in the population, which were expressed as the estimated graft years lost (eGYL) and estimated patient-years lost (ePYL). We also represented the cumulative mismatch penalty in eGYL per 100 patient-years (29). All analyses were performed using the R statistical language version 4.1.2 (30). Throughout this study we adhered to the Strengthening of Reporting of Observational Studies in Epidemiology (STROBE) guidelines.

## 3 Results

### 3.1 Patient population characteristics

After applying our exclusion criteria and matching recipients by paired-donor groups, a total of 105,608 DDKT recipients were identified for analysis (**Figure 1**). We matched a total of 31,518 DDKT recipients for the CMV D+/R+ and CMV D+/R- paired groups and 21,286 DDKT recipients for the CMV D-/R+ and CMV D-/R- paired groups. Age at the time of transplantation ranged from 18 to 87 years, with a median of 55 years. 62.8% of the total population was male, 31.8% identified as Black, and 84.9% identified as non-Hispanic. A large majority (87.2%) of DDKT recipients had received pre-KT dialysis and 37% had a diabetes diagnosis at the time of transplantation. HLA mismatches were common; 27.7% had 4 mismatches and 31.5% had 5 mismatches (**Table 1**).

**Table 1:** Patient population characteristics for paired-kidney analysis, 2011 to 2022.

	CMV D+/R+ (N=31518)	CMV D+/R- (N=31518)	CMV D-/R- (N=21286)	CMV D-/R+ (N=21286)	Total (N=105608)	p-value
Age						
Mean (SD)	53.9 (12.7)	52.6 (13.3)	52.2 (13.5)	53.4 (13.1)	53.1 (13.1)	<0.001
Median [Min, Max]	56.0 [18.0, 86.0]	54.0 [18.0, 86.0]	54.0 [18.0, 87.0]	55.0 [18.0, 84.0]	55.0 [18.0, 87.0]	
Sex						
Female	13454 (42.7%)	9756 (31.0%)	6802 (32.0%)	9272 (43.6%)	39284 (37.2%)	<0.001
Male	18064 (57.3%)	21762 (69.0%)	14484 (68.0%)	12014 (56.4%)	66324 (62.8%)	
Race						
Asian	2740 (8.7%)	700 (2.2%)	514 (2.4%)	1854 (8.7%)	5808 (5.5%)	<0.001
Black	11842 (37.6%)	8458 (26.8%)	5446 (25.6%)	7870 (37.0%)	33616 (31.8%)	
Multiracial	202 (0.6%)	264 (0.8%)	136 (0.6%)	126 (0.6%)	728 (0.7%)	
Native American or Alaska Native	336 (1.1%)	240 (0.8%)	136 (0.6%)	224 (1.1%)	936 (0.9%)	
Native Hawaiian or Pacific Islander	186 (0.6%)	42 (0.1%)	34 (0.2%)	112 (0.5%)	374 (0.4%)	
White or Caucasian	16212 (51.4%)	21814 (69.2%)	15020 (70.6%)	11100 (52.1%)	64146 (60.7%)	
Ethnicity						
Hispanic	6368 (20.2%)	3644 (11.6%)	2084 (9.8%)	3872 (18.2%)	15968 (15.1%)	<0.001
Non-Hispanic or unknown	25150 (79.8%)	27874 (88.4%)	19202 (90.2%)	17414 (81.8%)	89640 (84.9%)	
Dialysis (pre-transplant)						
Yes	18098 (85.0%)	18918 (88.9%)	27056 (85.9%)	27920 (88.7%)	91992 (87.2%)	<0.001
No	3188 (15.0%)	2368 (11.1%)	4426 (14.1%)	3562 (11.3%)	13544 (12.8%)	
Diabetic (Type 1 or Type 2)						
Yes	7412 (34.8%)	7906 (37.1%)	11356 (36.1%)	12338 (39.2%)	39012 (37.0%)	<0.001
No	13874 (65.2%)	13380 (62.9%)	20126 (63.9%)	19142 (60.8%)	66522 (63.0%)	
Missing	0 (0%)	0 (0%)	0 (0%)	2 (0.0%)	2 (0.0%)	
Creatinine (milligrams/deciliter)						
Mean (SD)	8.30 (3.74)	8.19 (3.84)	8.14 (3.86)	8.26 (3.74)	8.23 (3.79)	<0.001
Median [Min, Max]	7.84 [0.370, 32.1]	7.65 [0.400, 30.9]	7.60 [0.310, 32.4]	7.81 [0.100, 26.5]	7.73 [0.100, 32.4]	
Missing	154 (0.7%)	166 (0.8%)	196 (0.6%)	186 (0.6%)	702 (0.7%)	
Number of HLA mismatches						
0	1670 (5.3%)	1942 (6.2%)	1434 (6.7%)	1208 (5.7%)	6254 (5.9%)	<0.001
1	390 (1.2%)	380 (1.2%)	258 (1.2%)	284 (1.3%)	1312 (1.2%)	
2	1456 (4.6%)	1578 (5.0%)	1096 (5.1%)	1054 (5.0%)	5184 (4.9%)	
3	4124 (13.1%)	4512 (14.3%)	3186 (15.0%)	3052 (14.3%)	14874 (14.1%)	
4	8750 (27.8%)	8708 (27.6%)	5970 (28.0%)	5800 (27.2%)	29228 (27.7%)	
5	10238 (32.5%)	9850 (31.3%)	6366 (29.9%)	6764 (31.8%)	33218 (31.5%)	
6	4784 (15.2%)	4420 (14.0%)	2904 (13.6%)	3036 (14.3%)	15144 (14.3%)	
Missing	106 (0.3%)	128 (0.4%)	72 (0.3%)	88 (0.4%)	394 (0.4%)	
Waitlist Days						
Mean (SD)	860 (834)	872 (842)	858 (839)	875 (863)	866 (846)	0.0505
Median [Min, Max]	630 [0, 8550]	645 [0, 7320]	632 [0, 7700]	639 [0, 12500]	636 [0, 12500]	

### 3.2 One year post-transplant risks due to CMV D+/R- serostatus

**Figure 2:**
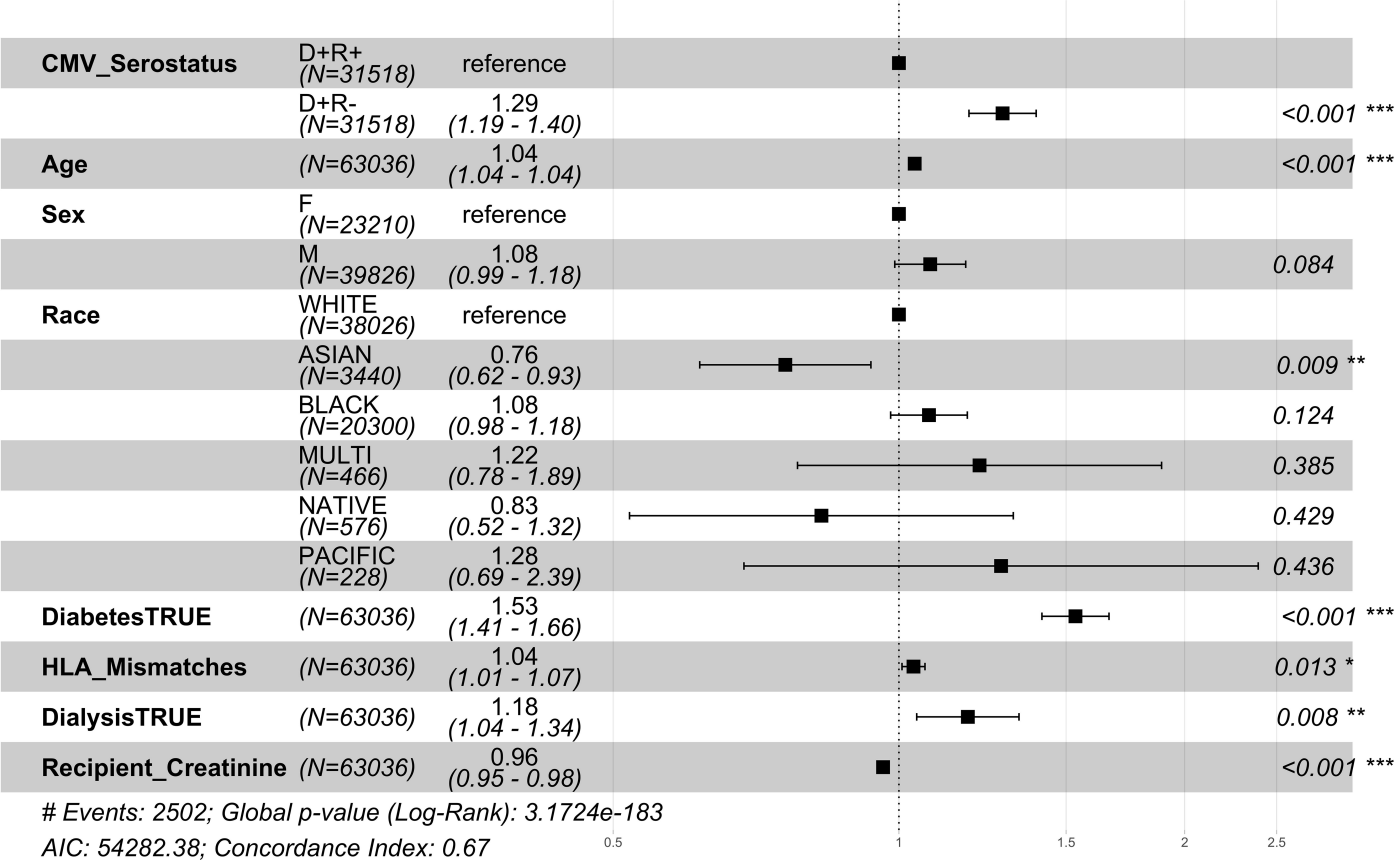
1 year post-transplant mortality hazard ratios for CMV donor-matched recipients. Here we provide adjusted hazard ratio forest plots, comparing the all-cause mortality of the two donor-positive groups, CMV donor positive/recipient negative [CMV D+/R-] group versus the CMV donor positive/recipient positive [CMV D+/R+] (reference). The hazard ratios for the eight risk covariates are provided. The p-value notation is as follows: p < 0.05 *, p < 0.01 **, p < 0.001 ***.

**Figure 3:**
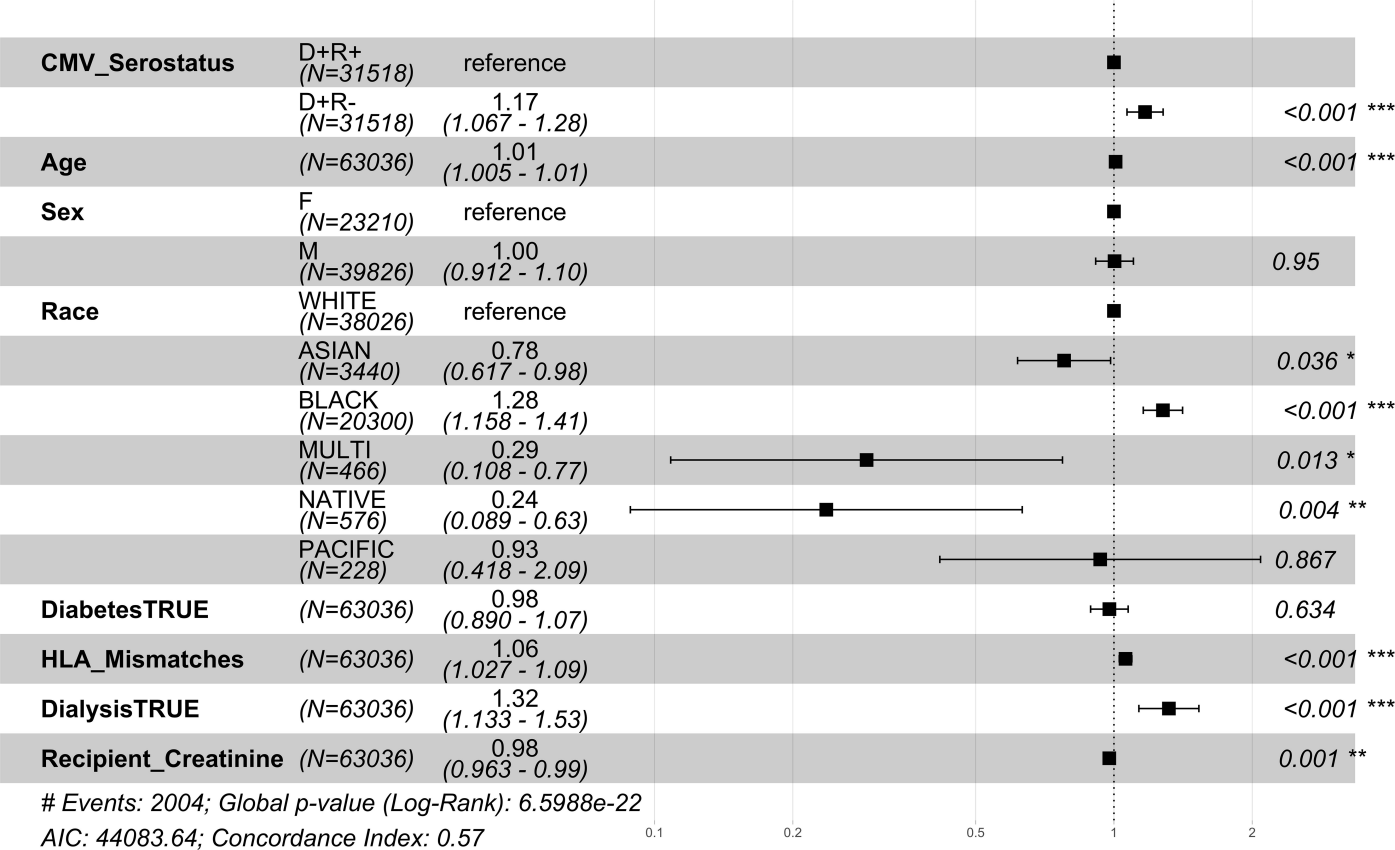
1 year post-transplant graft failure hazard ratios for CMV seropositive donor-matched recipients. Here we provide hazard ratio forest plots, comparing all-cause graft failure in the two donor-positive groups, CMV D+/R- and CMV D+/R+ (reference). The hazard ratios for the eight risk covariates are provided. The p-value notation is as follows: p < 0.05 *, p < 0.01 **, p < 0.001 ***.

Among the CMV D+/R- DDKT recipients, the hazard ratio for 1 year mortality and 1 year graft loss was increased by 29% and 17% respectively compared to CMV D+/R+ DDKT recipients (**Figures 2**, **3**). KM curves at 1 year post-DDKT for patient and graft survival are depicted in **Supplementary Figure 1**, respectively. Significant p-values were observed for patient (p<0.001) and graft survival (p=0.0045), with lower survival among CMV D+/R- compared to DMV D+/R+ recipients. Covariates associated with a higher risk of 1 year mortality in the CMV D+/R- DDKT recipients included greater age, male sex, Black race, diabetes, and pre-transplant dialysis. While not reaching statistical significance, the total number of HLA mismatches trended towards an association with a higher risk of 1 year post-transplant mortality. Because creatinine levels were very high in this population, lower creatinine at the time of DDKT was associated with a slightly decreased risk of mortality (HR=0.96; CI=0.95-0.98, p-value <0.001) and graft failure at 1 year (HR=0.98; CI=0.96-0.99, p-value<0.001). Factors that were associated with a higher relative risk for 1 year graft loss included greater age, Black race (compared to White), pre-transplant dialysis, the total number of HLA mismatches, and a higher creatinine level at the time of DDKT.

### 3.3 One year post-transplant risks due to CMV D-/R+ serostatus

Among the CMV D-/R+ DDKT recipients, the 1 year mortality and 1 year graft loss increased by 5% and 14% respectively compared to matched CMV D-/R- DDKT recipients (**Supplementary Figure 2**). We observed no significant differences in mortality; however, the graft failure hazard ratio was statistically higher (HR=1.14; CI=1.01-1.3, p-value=0.02) in the CMV D-/R+ DDKT recipients. KM curves demonstrating lower patient and graft survival at 1 year post-transplant and CMV D-/R+ compared to CMV D-/R- DKKT are shown in **Supplementary Figure 3**, with survival p-values of 0.048 and 0.012, respectively. Covariates associated with a higher relative risk for 1 year graft loss included greater age, Black race (compared to White), and pre-transplant dialysis. Due to this observation, we only analyzed the long-term effects of CMV D+/R- mismatching.

### 3.4 One year post-transplant mortality risk due to CMV D+/R- serostatus stratified by recipient age

**Figure 4:**
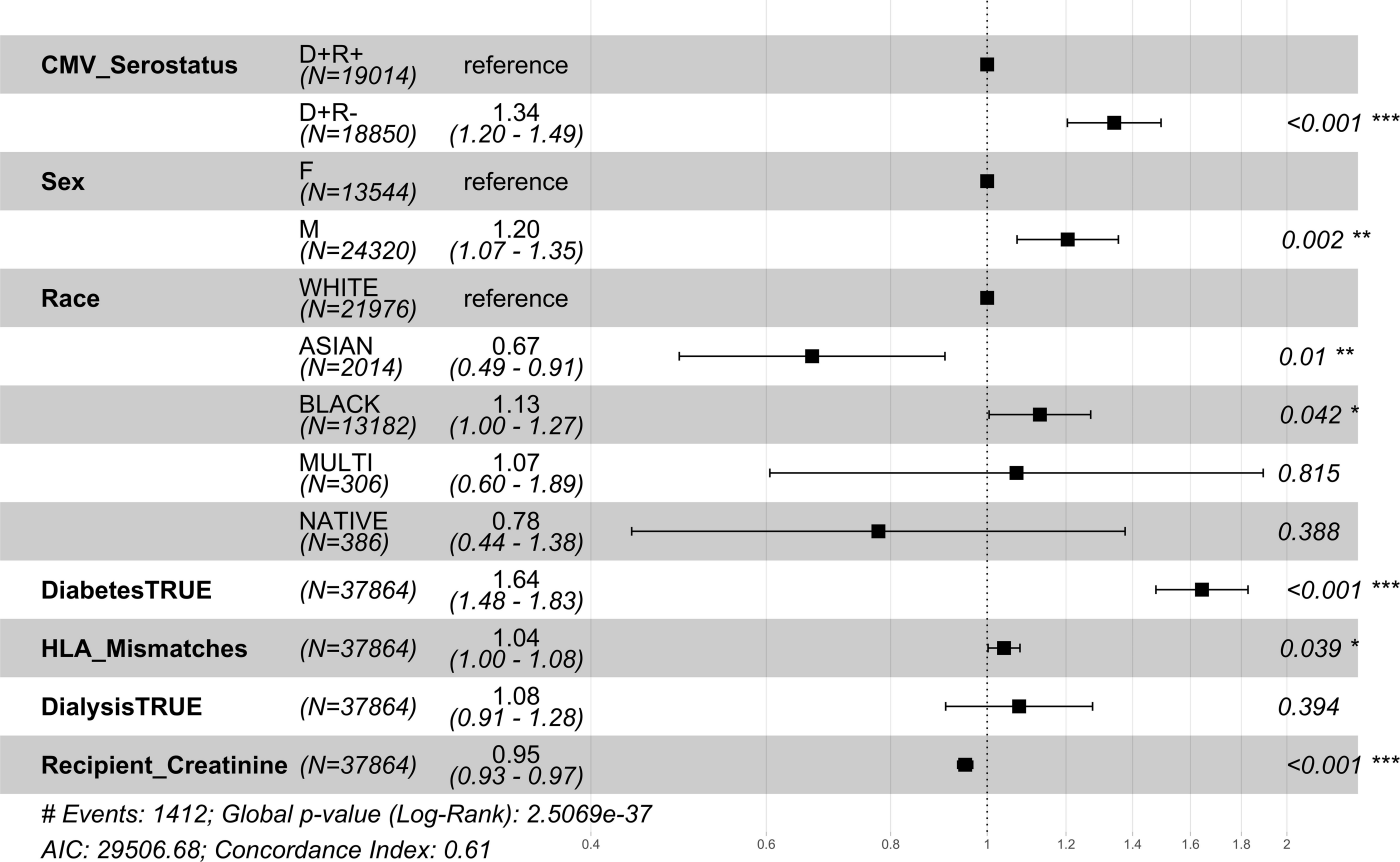
1 year post-transplant mortality hazard ratios for middle-aged CMV seropositive donors-matched recipients. Here we provide hazard ratio forest plots, comparing the mortality of the two middle-aged donor-positive groups. Middle-aged recipients were grouped according to their age at the time of transplant (41-64 years). The p-value notation is as follows: p < 0.05 *, p < 0.01 **, p < 0.001 ***.

**Figure 5:**
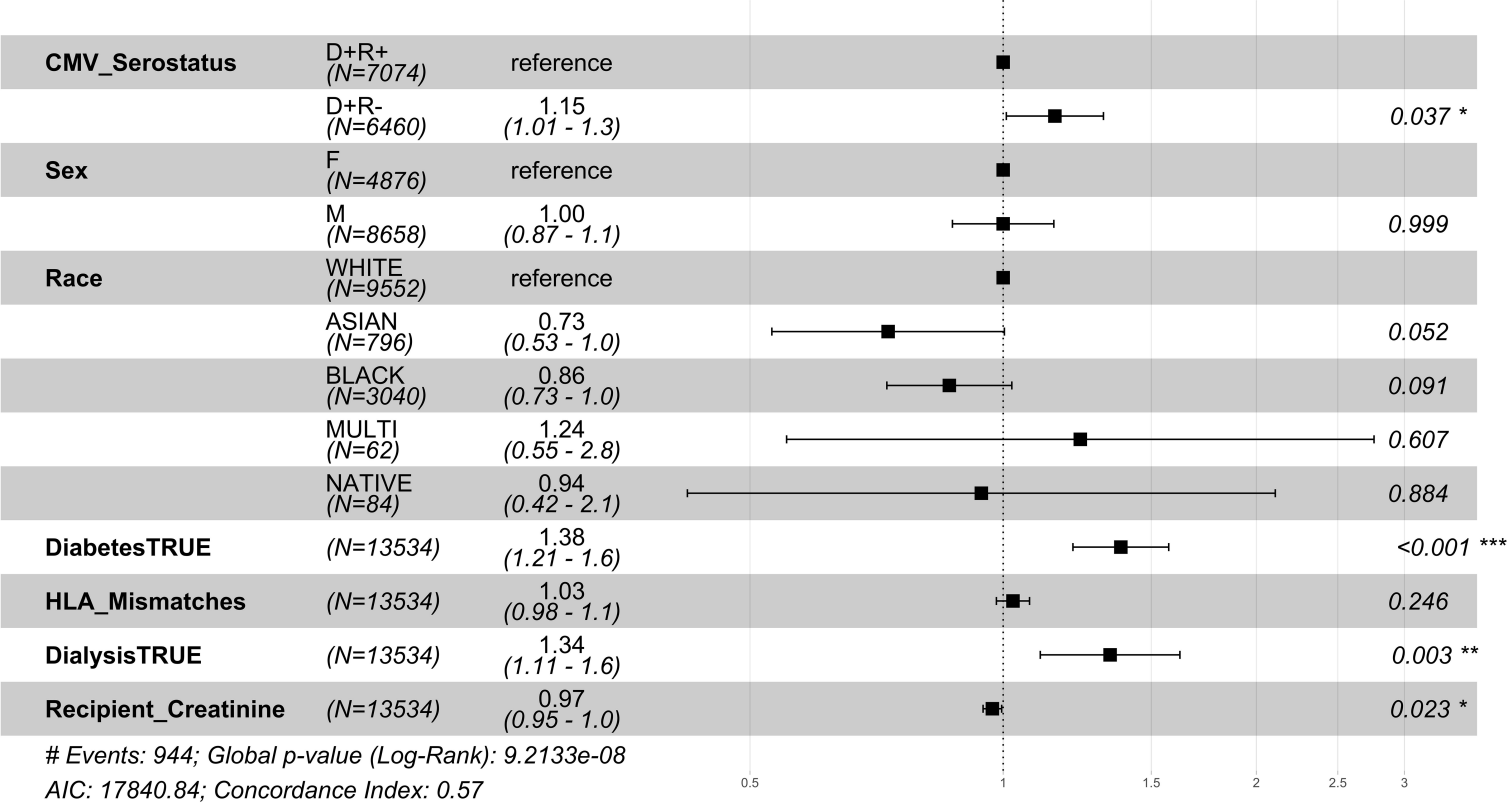
1 year post-transplant mortality hazard ratios for older CMV seropositive donors-matched recipients. Here we provide hazard ratio forest plots, comparing the mortality of the two older donor-positive groups. Older recipients were grouped according to their age at the time of transplant (≥ 65 years). The p-value notation is as follows: p < 0.05 *, p < 0.01 **, p < 0.001 ***.

The relative risk of 1 year mortality was higher amongst middle-aged (41-64 years) CMV D+/R- DDKT recipients (HR=1.34; CI=1.20-1.49, p-value<0.001) compared to middle-aged CMV D+/R+ middle-aged DDKT recipients (**Figure 4**). Similar but attenuated risk was seen among DDKT recipients ≥65 years (HR=1.15; CI=1.01-1.13, p-value=0.037, **Figure 5** comparing CMV D+/R- to CMV D+/R+). The impact of CMV D+/R- serostatus mismatching was not statistically significant (HR=1.182; CI=0.85-1.67, p-value=0.3457) among younger DDKT recipients (18-40 years). Similar trends in the risk coefficients of the covariates were observed. KM survival curves and risk tables were generated at 10 years post-KT for each age-stratified serostatus group and are provided in **Supplementary Figure 4**.

### 3.5 One year post-transplant mortality risk due to diabetes and dialysis

Next, we stratified the 1 year risk of mortality and graft loss by diabetes mellitus and pre-transplant dialysis status. At 1 year post-transplant, we found an 85% increase in the relative risk of mortality in patients with diabetes (N=23,720, HR=1.85; CI=1.7-2.0, p-value<0.001, **Supplementary Figure 5A**) as compared to those with no diabetes. We also found a 16% increase in the relative risk of mortality in CMV D+ patients on dialysis at the time of DDKT (N=55,042, HR=1.16; CI=1.02-1.31, p-value=0.023, **Supplementary Figure 5B**) as compared to those who did not receive dialysis prior to DDKT. Both observations were consistent with the previous risk-adjusted models.

### 3.6 Ten year risk due to CMV D+/R- serostatus

**Figure 6:**
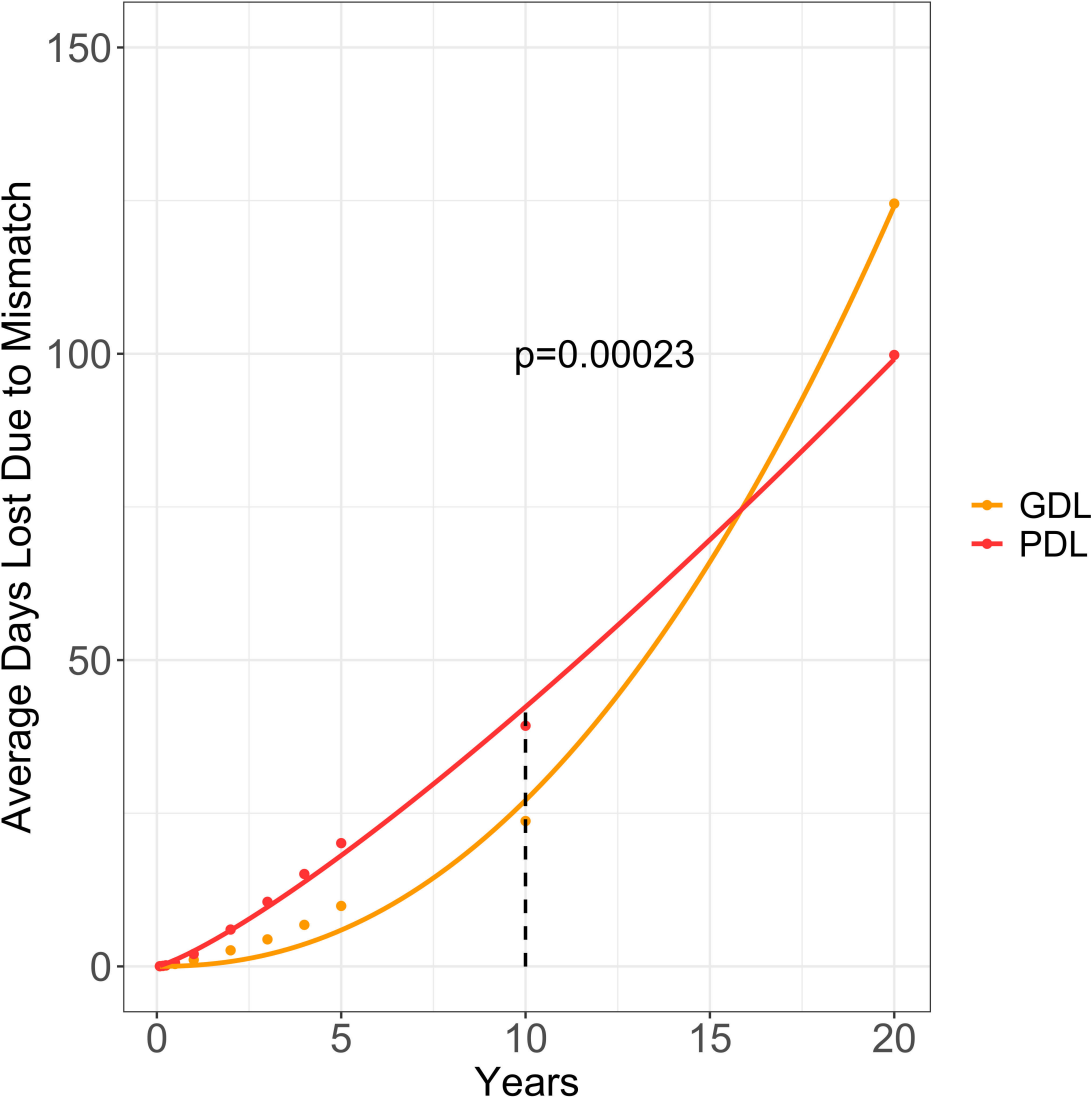
Estimated graft and patient days lost due to CMV donor-positive mismatching. This graph provides the average graft days lost (aGDL/patient) and average patient days lost (aPDL/patient) due to CMV mismatching for CMV donor-positive/recipient-negative (CMV D+/R-) mismatched DDKT recipients. All values from 1 to 10 years were calculated by subtracting the area under the CMV D+/R- Kaplan-Meier (KM) curve from the area under the CMV D+/R+ KM curve. Using parametric survival regression, we extrapolated the KM curves to 20 years and calculated the average graft days lost (orange) and average patient days lost (red). Graft Days Lost (GDL); Patient Days Lost (PDL).

10 year KM survival curves and risk tables were provided for the CMV D+ paired cohort (**Supplementary Figure 6**) and the CMV D- paired cohort (**Supplementary Figure 7**). At 10 years post-DDKT, the observed graft days lost per patient was 23.7 days and loss of patient days was 39.3 days. Extrapolating CMV D+ cohorts to 20 years post-DDKT, the estimated graft days lost per patient was 125 days and the estimated patient days was 100 days. Using linear approximation, we calculated the cumulative penalty for CMV D+/R- mismatching to provide a total estimated graft years lost of 16,146 and patient years lost as 12,943 among the CMV D+/R- population (**Figure 6**). With 47,368 CMV D+/R- mismatches over 20 years, we surveyed 947,360 total patient years (PY). The estimated graft years lost per 100 PY was 1.7 and the estimated patient years lost per 100 PY was 1.36.

## 4 Discussion

To the best of our knowledge, this is the largest paired DDKT study to report the cumulative risks of CMV D+/R- mismatching across the estimated lifespan of DDKT recipients and the CMV D+/R- population. Leveraging the SRTR, we demonstrated that CMV D+/R- serostatus is significantly and independently associated with an increased risk of graft loss and mortality. Importantly, we discovered a profound increase in mortality in middle-aged CMV D+/R- DDKT recipients compared with middle-aged CMV D+/R+ DDKT recipients. While older CMV D+/R- DDKT recipients were still at a higher 1 year relative risk for post-transplant mortality compared with older CMV D+/R+ DDKT recipients, the effect was attenuated. Herein we present a novel approach to estimating the cumulative penalty of CMV D+/R- mismatching over the lifespan of the DDKT recipient and across the affected population, with an average of 125 estimated graft and 100 estimated patient days lost per patient. The current kidney allocation policy in the US does not include CMV serodirected allocation, however, this analysis advances our understanding of the magnitude of CMV D+/R- serostatus mismatching and helps to bring this problem into clinical context.

Our results demonstrating the negative impact of CMV D+/R- mismatch on graft and patient survival are consistent with prior studies that link CMV D+/R- mismatch with poor graft and patient survival in kidney (18–20), liver (31), and thoracic (32) transplant recipients, but expand prior study designs by utilizing a paired-donor kidney cohort. Fewer studies have used the paired-donor approach. Utilizing the OPTN data, Leaphorn and colleagues created a paired kidney cohort of 52,394 DDKT recipients and demonstrated a 21% greater risk for all-cause mortality and a 47% greater risk for infection-related mortality in CMV D+/R- DDKT recipients compared with D+/R+ DDKT recipients (19). The difference between this study and Leaphorn et al. is that we analyzed a much larger cohort with a more comprehensive dataset. The importance of CMV prevention is highlighted by a recent study in a different transplant population of haploidentical hematopoietic cell transplant recipients, where CMV reactivation was noted to be the major driver of mortality (33). While it is likely that short-term mortality in CMV D+/R- DDKT recipients in our study may be directly related to CMV disease, the exact mechanisms underlying long-term patient and graft mortality remain unclear and may be attributable to the indirect effects of CMV infection. These indirect pathological effects may include increased risk for thrombotic events, secondary infection, and persistent systemic inflammation secondary to sustained low-level CMV replication which eventually leads to decreased patient and graft survival (1, 19, 31, 34–38). Future studies are needed to define the biological mechanisms responsible for the adverse long-term outcomes in CMV D+/R- DDKT recipients.

Another important finding in this study was the increased risk of mortality observed in middle-aged CMV D+/R- DDKT recipients who are likely to live longer and experience a higher penalty from CMV serostatus mismatch. The effect of CMV D+/R- mismatch was profound concerning mortality in middle-aged DDKT recipients who had significantly higher risk than CMV D+/R+ middle-aged DDKT recipients. Among older adults when stratified by CMV D/R status, older CMV D+/R+ DDKT recipients were at an attenuated risk compared to CMV D+/R- older DDKT recipients. Middle-aged and older CMV D+/R- DDKT recipients may gain additional benefit from CMV serostatus matching policies and immune-based strategies such as CMV vaccination or CMV-specific antibodies (39, 40).

The duration for CMV prophylaxis in transplant centers in the US is guided by CMV serostatus (5, 41) and does not account for the age of the recipient. Given that older CMV D+/R- DDKT recipients were at a much higher risk of graft loss after stopping CMV prophylaxis, they may benefit from a longer duration of prophylaxis beyond six months or the use of a hybrid approach of prophylaxis and pre-emptive therapy that includes the serial monitoring of the CMV viral load following discontinuation of prophylaxis (42).

Because CMV prevention and treatment strategies are far from effective, the importance of our findings and a possible need for CMV serodirected allocation is further highlighted by a recent study that reports a significant increase in the proportion of CMV D+/R- SOTs across all organs (43). The projected proportion of CMV D+/R- SOTs will increase significantly by 2040, resulting in an estimated predicted prevalence of 20.6% for CMV D+/R- DDKT recipients, driven more a disproportionate decrease in CMV seroprevalence among SOT candidates rather than an increase in CMV D+ (43). Altering the national allocation policy by matching SOT recipients by donor CMV serostatus could significantly improve survival and reduce the costs associated with hospitalization and the treatment of CMV infection and its many complications, and has been successfully demonstrated in a pilot study conducted by three kidney transplant centers in Oregon (44). Importantly, this CMV seromatching policy did not prolong wait times and demonstrated a significant increase in low-risk CMV D-/R- offers while observing a significant decrease in high-risk CMV D+/R- DDKT recipients (44). Following this policy change was a dramatic reduction in the rates of CMV infection, the cumulative duration of antiviral prophylaxis, and the costs related to prophylaxis and treatment (44). Subsequent studies have shown that CMV serostatus matching appeared both cost-saving and was associated with prolonged patient survival, provided the recipients’ waitlist time did not exceed 30 months (45). The survival advantage of CMV seromatching has been shown in allogeneic hematopoietic stem cell recipients, resulting in the widespread use of CMV seromatching in these recipients (11, 46, 47). Further studies are needed in SOT recipients to assess the impact of CMV serodirected allocation on patient waitlist time, considering other significant risk factors such as HLA matching, ABO compatibility, and Kidney Donor Profile Index score.

The strengths of our study include a large cohort size of 105,608 DDKT recipients with little missing data who adequately represent the DDKT population in the US. By conducting a paired kidney analysis, we reduced biases due to differences in donor characteristics. We acknowledge several significant limitations inherent in a registry-based, retrospective study. These risk models are dependent on the accuracy of event reporting in the SRTR database and the Social Security Death Index file. Our analysis and the covariates used in this study are limited to the variables collected in the SRTR database. We could not include all known risk factors associated with end-stage kidney disease or with graft or patient survival, including major adverse cardiac events, non-death censored graft failures, other infections, or other complications that could have a substantial impact on long-term graft and patient survival (48, 49). Understanding these confounding factors is important to interpreting and contextualizing the results provided herein (50). Donor and recipient’s antiviral drug dosages, the duration of antiviral prophylaxis or antiviral therapy, and reinfections are not captured in this study. We were unable to compare recipients who did and did not receive CMV prophylaxis with pre-emptive therapy; however, we specifically targeted a study period between 2011 and 2022 during which all major CMV prevention and prophylaxis treatments were widely in use. Also, the SRTR does not contain data on the CMV viral loads post-DDKT, so we could not capture CMV seroconversion or analyze the impact of new detectable and quantifiable CMV infection and reactivations. The SRTR does not capture variations in immune suppressive regimens that could impact the occurrence of CMV infections. These confounders limited the interpretation of these results and should also be carefully considered. Additionally, our donor-paired analysis, we did not directly compare the high-risk CMV D+/R- mismatch group with the lowest-risk CMV D-/R- group. Lastly, the accuracy of extrapolating patient and graft survival curves relies on model assumptions and changing conditions, such as long-term patient survival rates, disease etiology, and other advancements in patient care and treatment. Thus, the extrapolation results are only applicable to this cohort.

In summary, we have demonstrated a significant penalty that CMV D+/R- mismatching confers on long-term patient and graft survival, especially in middle-aged DDKT recipients in an era where CMV preventive or pre-emptive strategy is standard practice. The long-term impact of CMV D+/R- mismatching on graft survival and mortality suggests a critical need to further investigate the feasibility and efficacy of CMV matching organ allocation strategies at a larger scale across multiple sites. The greater impact of CMV D+/R- mismatching on middle-aged and older DDKT recipients also supports the need for future randomized clinical trials comparing the extended duration of antiviral prophylaxis or hybrid prophylaxis and pre-emptive therapy approaches compared to the standard six-month duration of prophylaxis. While the long-term impact of CMV D+/R- mismatching in SOT recipients has well-established consequences on morbidity and mortality, safer CMV therapies and innovative strategies to reduce mismatching hold promise for mitigating the known risks of CMV mismatching.

## Data availability statement

Publicly available datasets were analyzed in this study. This data can be found here: https://www.srtr.org/.

## Ethics statement

The studies involving humans were approved by Colorado Multiple Institutions Review Board. The studies were conducted in accordance with the local legislation and institutional requirements. Written informed consent for participation was not required from the participants or the participants’ legal guardians/next of kin in accordance with the national legislation and institutional requirements.

## Author contributions

MA: Conceptualization, Funding acquisition, Resources, Writing – original draft, Writing – review & editing. JS: Conceptualization, Investigation, Resources, Supervision, Writing – review & editing. BK: Conceptualization, Funding acquisition, Investigation, Resources, Supervision, Writing – review & editing. AW: Conceptualization, Supervision, Writing – review & editing. KE: Conceptualization, Funding acquisition, Resources, Supervision, Writing – review & editing. JM: Conceptualization, Data curation, Formal analysis, Investigation, Methodology, Project administration, Software, Supervision, Validation, Writing – original draft, Writing – review & editing.
