# Supplementary material for: Patient years lost due to cytomegalovirus serostatus mismatching in the scientific registry of transplant recipients
Source: Front Immunol. 2024 Jan 9;14:1292648. doi: 10.3389/fimmu.2023.1292648 (PMC10803440; doi:10.3389/fimmu.2023.1292648)
Supplement: Supplementary file 1 [file DataSheet_1.docx]

**Supplementary Appendix**

Supplement to**:** Patient Years Lost Due to Cytomegalovirus Serostatus Mismatching in the Scientific Registry of Transplant Recipients

This appendix has been provided by the authors to give readers additional information about the work.

**Supplementary appendix for:**

*Patient Years Lost Due to Cytomegalovirus Serostatus Mismatching in the Scientific Registry of Transplant Recipients*

**Contents:**

**Supplemental Figure 1.** 1 Year Post-transplant Patient and Graft Survival Curves for CMV Seropositive Donor (CMV+) Pairs. 3

**Supplemental Figure 2.** 1 Year Post-Transplant Patient and Graft Survival Hazard Ratios for CMV Seronegative Donor (CMV-) Pairs. 4

**Supplemental Figure 3.** 1 Year Post-transplant Patient and Graft Survival Curves for CMV Seronegative (CMV-) Donor Pairs. . 5

**Supplemental Figure 4.** 10 Year Post-transplant Survival Curves for CMV Seropositive Donor (CMV+) Pairs Stratified by Age. 6

**Supplemental Figure 5.** 1 Year Post-transplant Mortality Hazard Ratio for CMV Seropositive Donor (CMV+) Pairs by Diabetes and Dialysis Status. 7

**Supplemental Figure 6.** 10 Year Post-transplant Patient and Graft Survival Curves for CMV Seropositive Donor (CMV+) Pairs. 8

**Supplemental Figure 7.** 10 Year Post-transplant Patient and Graft Survival Curves for CMV Seronegative Donor (CMV-) Pairs. 9

**Supplemental Figure 1. 1 Year Post-transplant Patient and Graft Survival Curves for CMV Seropositive Donor (CMV+) Pairs.** A) Kapan-Meier patient survival curves, where the ‘CMV Recipient Seronegative (CMV REC-)‘ group (blue) demonstrated a decreased (p<0.001) 1 year post-transplant survival compared with ‘CMV Recipient Seropositive (CMV REC+)’ group (yellow). B) Kapan-Meier graft survival curves, where the ‘CMV REC-‘group (blue) demonstrated a decreased (p<0.001) 1 year post-transplant graft survival.

**
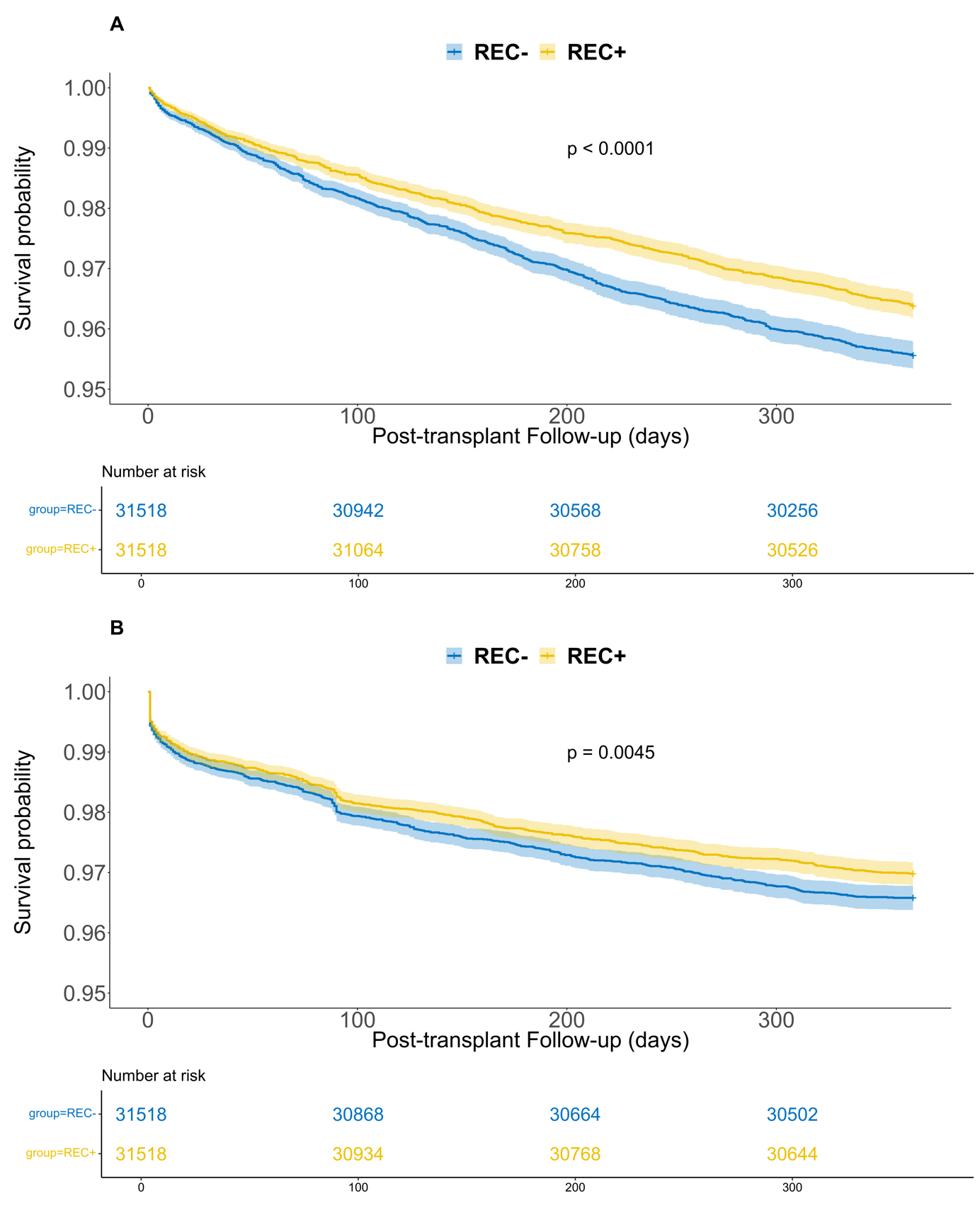
**

**Supplemental Figure 2. 1 Year Post-Transplant Patient and Graft Survival Hazard Ratios for CMV Seronegative Donor (CMV-) Pairs.**

**
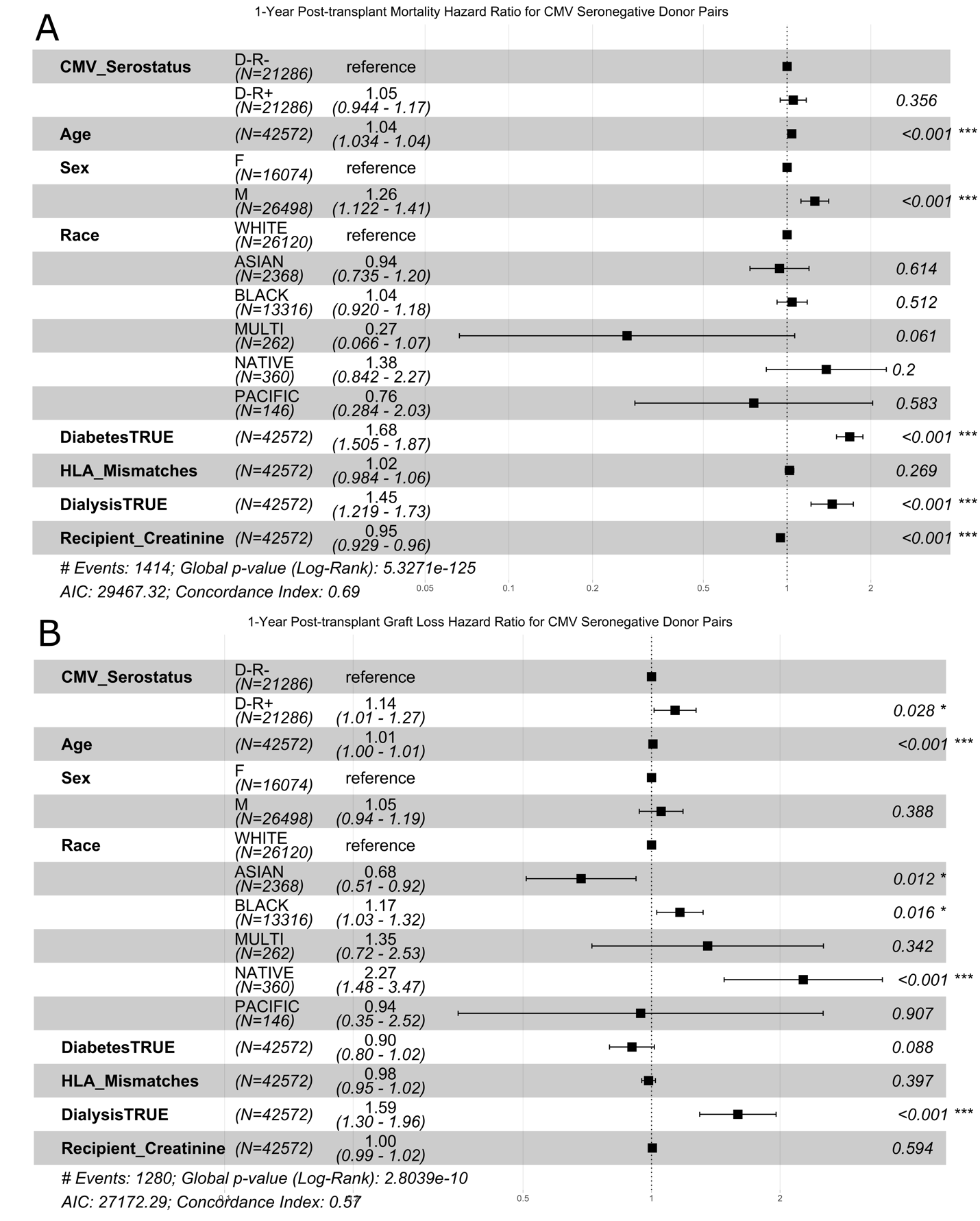
**

**Supplemental Figure 3. 1 Year Post-transplant Patient and Graft Survival Curves for CMV Seronegative (CMV-) Donor Pairs.** A) Kapan-Meier patient survival curves, where the ‘CMV Seropositive Recipient (CMV REC+)‘ group (blue) demonstrated a slightly decreased (p<0.048) 1 year post-transplant survival compared with CMV Seronegative Recipient ‘(CMV REC-)’. B) Kapan-Meier graft survival curves, where the ‘CMV REC+‘ group’ (blue) demonstrated a decreased (p<0.012) 1 year post-transplant graft survival. **
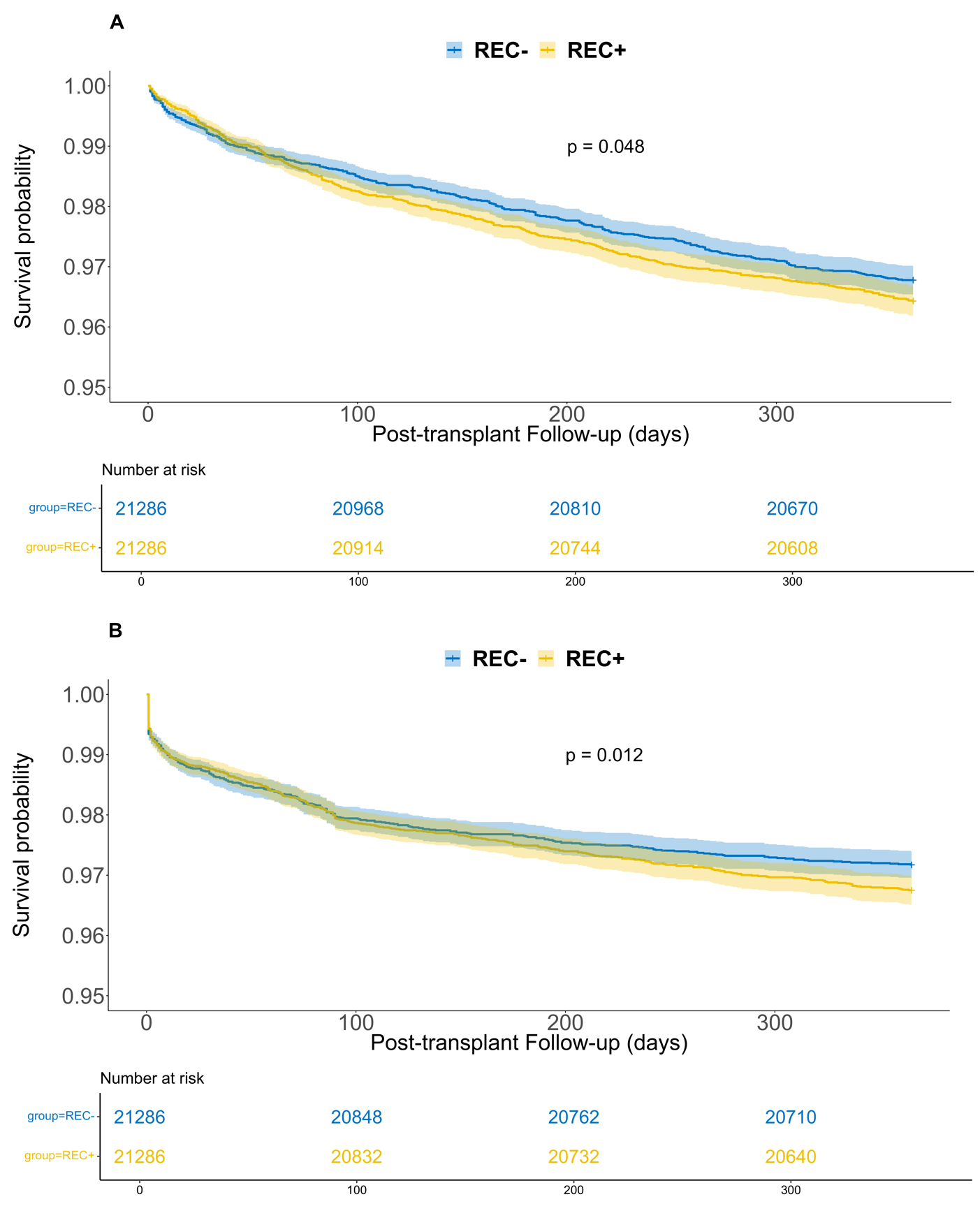
**

**Supplemental Figure 4. 10 Year Post-transplant Survival Curves for CMV Seropositive Donor (CMV+) Pairs Stratified by Age.**

**
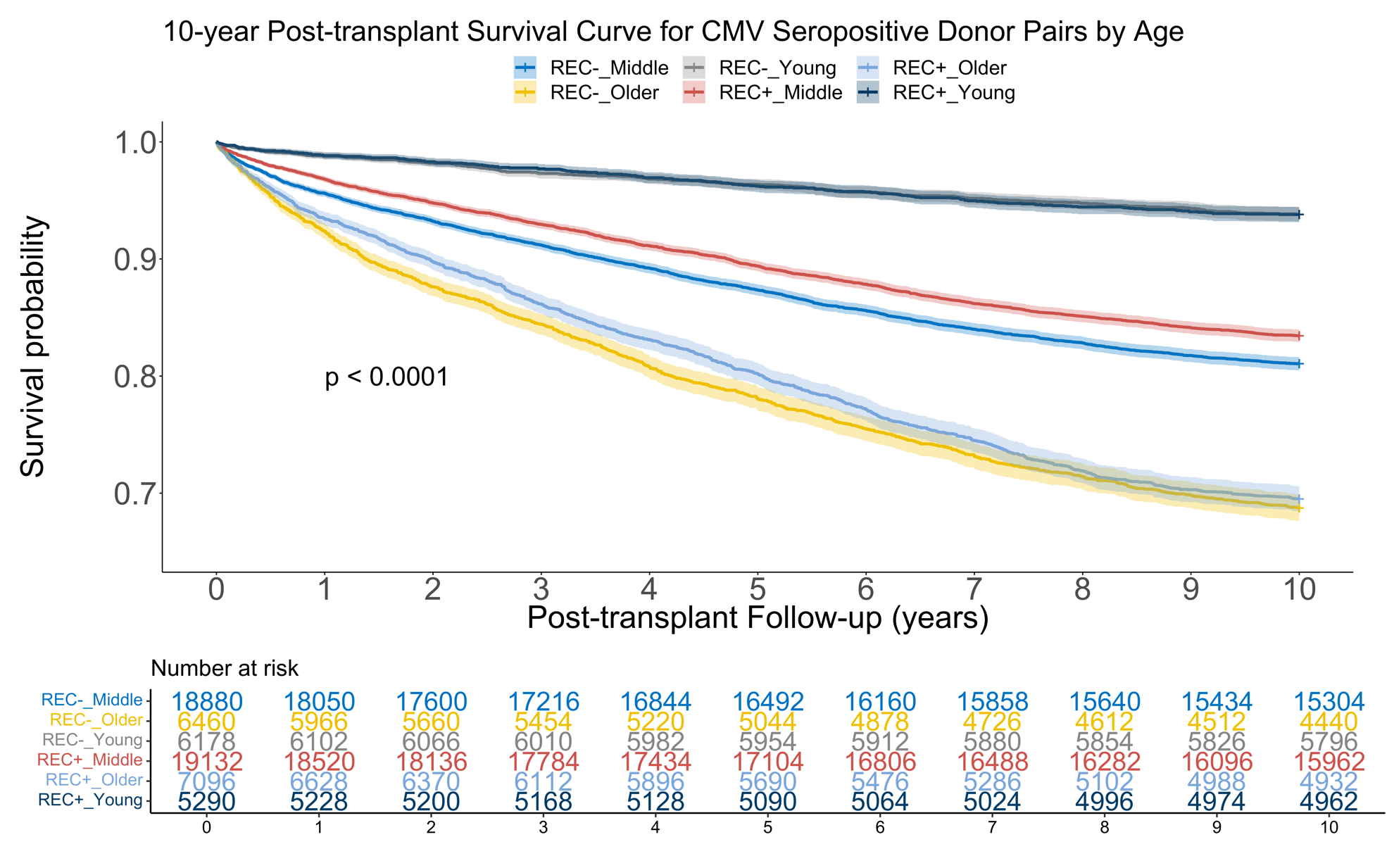
**

**Supplemental Figure 5. 1 Year Post-transplant Mortality Hazard Ratio for CMV Seropositive Donor (CMV+) Pairs by Diabetes and Dialysis Status.**

**
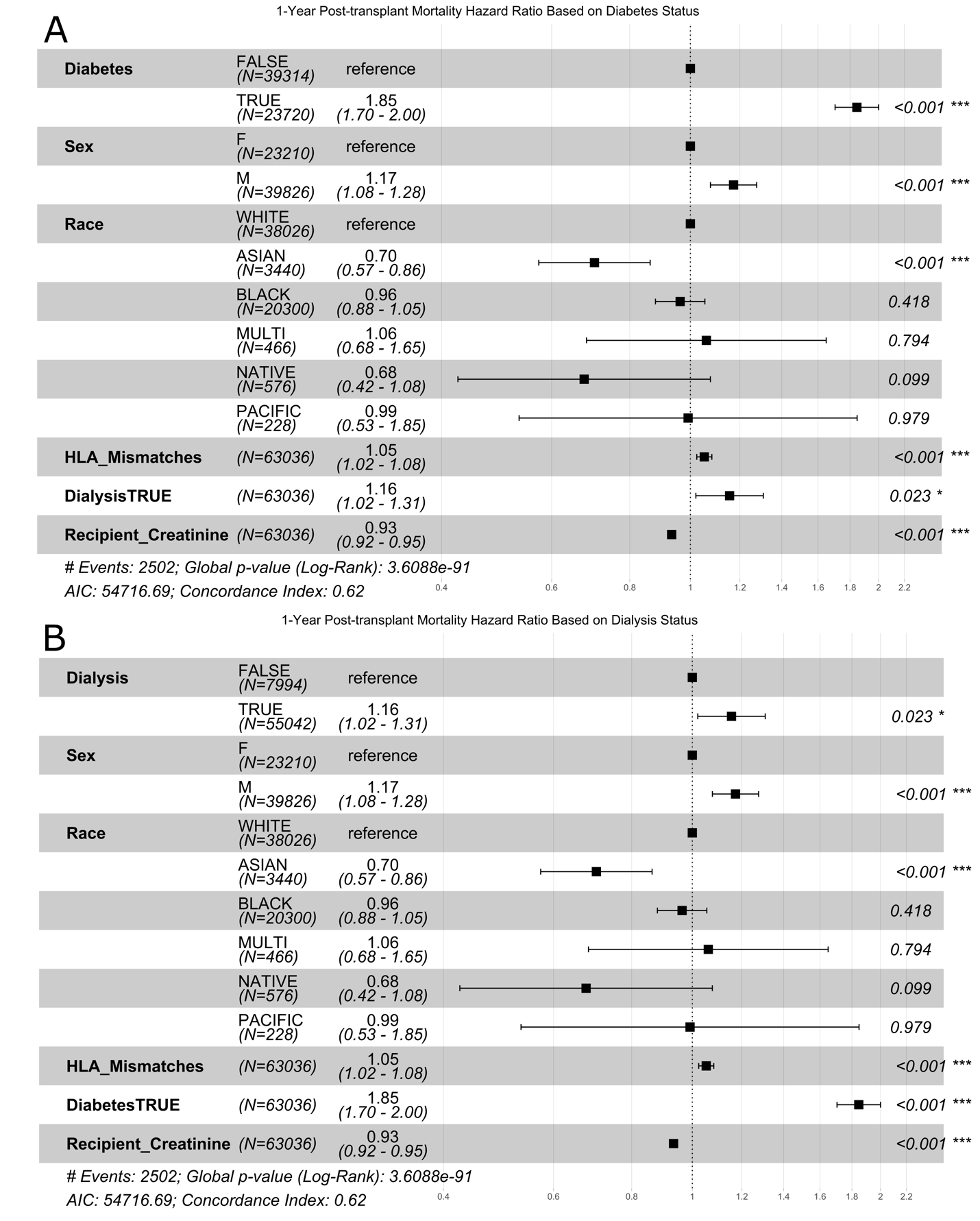
Supplemental Figure 6. 10 Year Post-transplant Patient and Graft Survival Curves for CMV Seropositive Donor (CMV+) Pairs.** A) Kapan-Meier patient survival curves, where the CMV Seropositive Recipient ‘CMV REC+‘ group (blue) demonstrated a decreased (p<0.0004) 10 years post-transplant survival compared with CMV Seronegative REC ‘CMV REC-‘ group (yellow). B) Kapan-Meier graft survival curves, where the ‘CMV REC+‘ group (blue) demonstrated a decreased (p=0.0053) 10 years post-transplant graft survival.

**
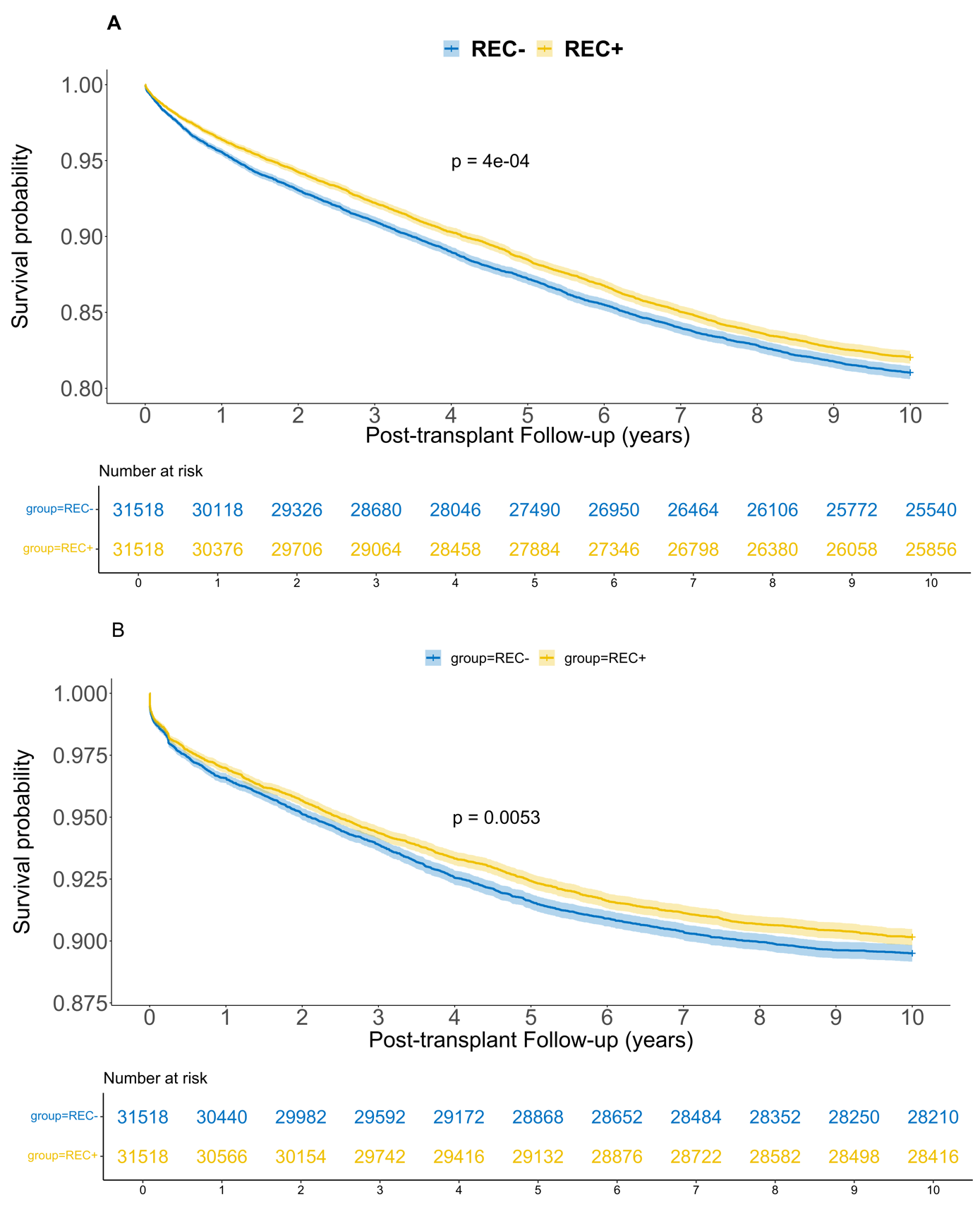
**

**Supplemental Figure 7. 10 Year Post-transplant Patient and Graft Survival Curves for CMV Seronegative Donor (CMV-) Pairs.** Kapan-Meier patient (A) and graft (B) survival curves from CMV- paired donor recipient groups [CMV seronegative recipient (CMV REC-) and CMV seropositive recipient (CMV REC+)]. No survival advantage was observed in these cohorts at 10 years post-transplant.

**
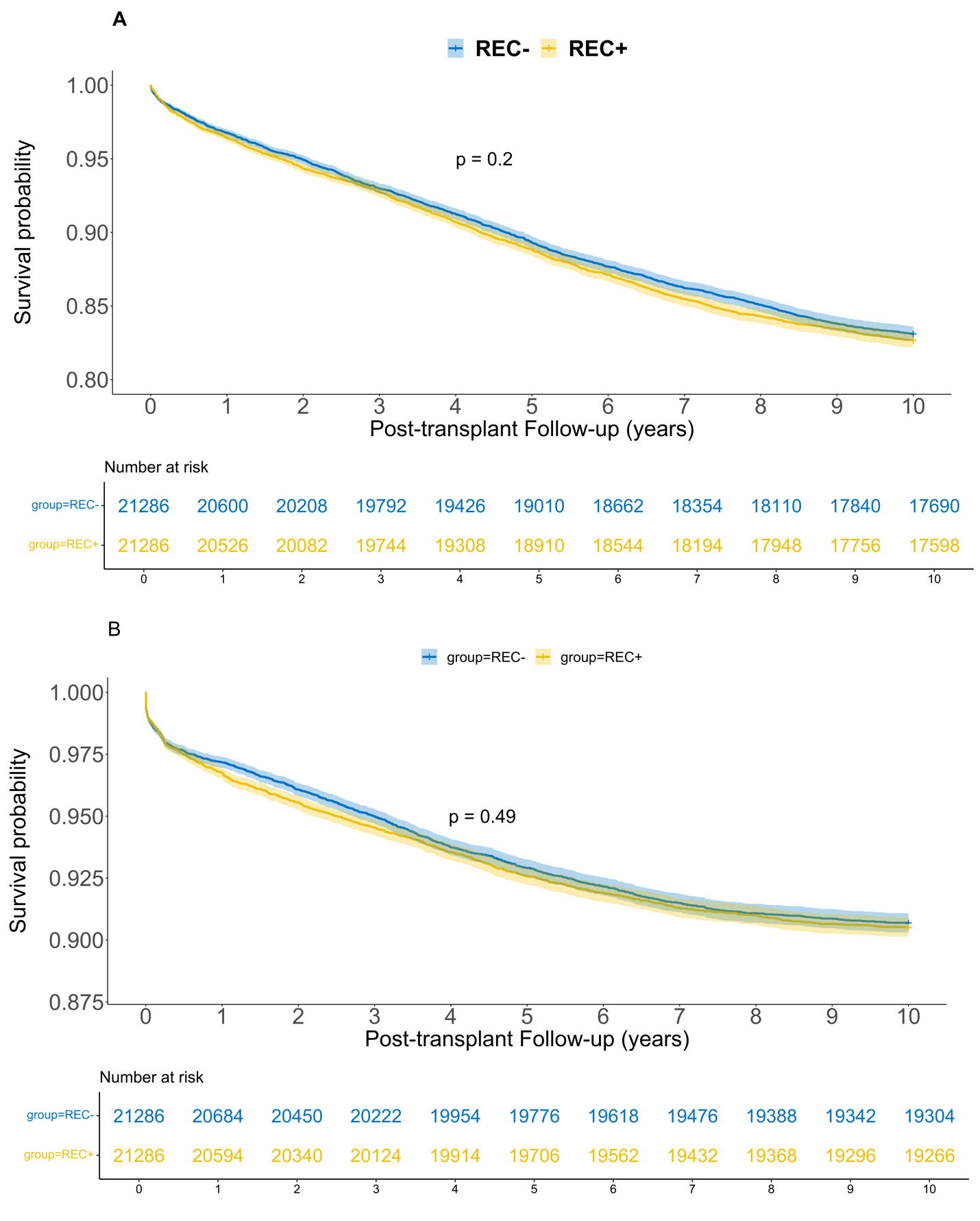
**
